# Generation and Analysis of a Mouse Intestinal Metatranscriptome through Illumina Based RNA-Sequencing

**DOI:** 10.1371/journal.pone.0036009

**Published:** 2012-04-27

**Authors:** Xuejian Xiong, Daniel N. Frank, Charles E. Robertson, Stacy S. Hung, Janet Markle, Angelo J. Canty, Kathy D. McCoy, Andrew J. Macpherson, Philippe Poussier, Jayne S. Danska, John Parkinson

**Affiliations:** 1 Program in Molecular Structure and Function, The Hospital for Sick Children, Toronto, Canada; 2 Division of Infectious Diseases, University of Colorado, Aurora, Colorado, United States of America; 3 Department of Molecular, Cellular and Developmental Biology, University of Colorado, Boulder, Colorado, United States of America; 4 Department of Molecular Genetics, University of Toronto, Toronto, Canada; 5 Department of Immunology, University of Toronto, Toronto, Canada; 6 Program in Genetics and Genomic Biology, The Hospital for Sick Children, Toronto, Canada; 7 Department of Mathematics and Statistics, McMaster University, Hamilton, Canada; 8 Department Klinische Forschung, University of Bern, Bern, Switzerland; 9 Sunnybrook Health Sciences Centre Research Institute, University of Toronto, Toronto, Canada; 10 Department of Medical Biophysics, University of Toronto, Toronto, Canada; 11 Department of Biochemistry, University of Toronto, Toronto, Canada; Cairo University, Egypt

## Abstract

With the advent of high through-put sequencing (HTS), the emerging science of metagenomics is transforming our understanding of the relationships of microbial communities with their environments. While metagenomics aims to catalogue the genes present in a sample through assessing which genes are actively expressed, metatranscriptomics can provide a mechanistic understanding of community inter-relationships. To achieve these goals, several challenges need to be addressed from sample preparation to sequence processing, statistical analysis and functional annotation. Here we use an inbred non-obese diabetic (NOD) mouse model in which germ-free animals were colonized with a defined mixture of eight commensal bacteria, to explore methods of RNA extraction and to develop a pipeline for the generation and analysis of metatranscriptomic data. Applying the Illumina HTS platform, we sequenced 12 NOD cecal samples prepared using multiple RNA-extraction protocols. The absence of a complete set of reference genomes necessitated a peptide-based search strategy. Up to 16% of sequence reads could be matched to a known bacterial gene. Phylogenetic analysis of the mapped ORFs revealed a distribution consistent with ribosomal RNA, the majority from Bacteroides or Clostridium species. To place these HTS data within a systems context, we mapped the relative abundance of corresponding *Escherichia coli* homologs onto metabolic and protein-protein interaction networks. These maps identified bacterial processes with components that were well-represented in the datasets. In summary this study highlights the potential of exploiting the economy of HTS platforms for metatranscriptomics.

## Introduction

Boosted by the advent of high through-put sequencing (HTS) platforms, metagenomics has emerged as a powerful approach for analyzing complex bacterial communities [1], [2]. Typically such studies focus on the random (shotgun) sequencing of DNA to define the relative abundance of genes within a community [3], [4]. Further inferences on community structure can be gleaned through 16S rRNA gene surveys which provide information on relative species abundance [5], [6]. As such, functional insights are limited to cataloguing genes within a sample, either through direct sequence identification (shotgun sequencing) or inference through reference genomes (16S rRNA gene surveys). Understanding the functional relationships within bacterial communities would be significantly enhanced by the analysis of bacterial gene/protein expression [7]. Previous metatranscriptome studies have relied on cDNA-RFLP and microarray approaches [8]–[10] and have been largely limited to the analysis of known genes. More recently, several groups have reported metatranscriptomic studies with HTS platforms [11]–[17]. These studies report several challenges for optimizing the yield of informative reads, HTS data analyses and presentation of these complex data.

Choice of HTS platform has a significant impact on the yield of informative reads. In most recent RNA HTS (RNA-Seq) studies, mRNA are mapped onto reference genomes for a single species (e.g. mouse, human) [18], [19]. For complex bacterial communities, such as the rodent or human intestine, complete sets of reference genomes are lacking. Furthermore, given the extensive sequence diversity that can occur even between related bacterial species, generation of a complete set of reference genomes is currently beyond current sequencing capabilities. To increase the probability of obtaining a significant match to previously identified sequences, metatranscriptomic studies have relied on the 454 Titanium (Roche) platform [12], [14], [20] which generates longer reads than the Illumina [21] and SOLiD (Applied Biosystems, Carlsbad, Ca) platforms. However, the SOLiD and Illumina platforms offer a significant improvement in read coverage over 454 for the same unit cost enhancing interest in their application to metatranscriptomics. One solution is to use a hybrid approach in which 454 is used first to assemble a series of reference DNA sequences onto which Illumina- or SOLiD-generated transcript data can be mapped [22]. A second factor affecting yields of informative reads is RNA sample preparation. In addition to purification of high quality RNA, it is desirable to reduce the proportion of ribosomal RNA (rRNA) sequences with commercial kits that remove 16S and 23S rRNA molecules providing enrichment for the high complexity mRNA [7]. However, questions remain about the effectiveness and potential bias of the rRNA removal approaches.

Bioinformatics analyses of metatranscriptomics datasets are an intensive area of research and development. Previous approaches have focused on matching sequences to gene families as defined by knowledgebases such as the Clusters of Orthologous Groups (COG), Gene Ontology (GO) and SEED resources [23]–[25]. Such annotation schemes provide high-level comparisons across broad functional categories. To provide more detailed, molecular level insights, recent studies are beginning to explore the use of biochemical pathway analyses [3], [26]. For example, the iPath tool [27] has been used to map reads onto metabolic pathways defined by the Kyoto Encyclopedia of Genes and Genomes (KEGG [28]) to produce an integrated view of the metabolic capabilities of an environmental sample [3]. With the availability of high quality protein interaction datasets, the opportunity exists to exploit these exceptional datasets as functionally coherent scaffolds on which to organize and interpret metatranscriptomic data. As such, these datasets offer the possibility to extend systems-based analyses beyond metabolism to capture additional bacterial subsystems such as protein complexes or signaling and transport pathways.

Here we are interested in exploring the feasibility of using the Illumina HTS platform to perform metatranscriptomics on a model gut microbiome. Our choice of experimental system is guided by the recent appreciation of the considerable role that commensal flora in the human gut play in human health and disease. For example, metagenomic surveys have already illuminated potential mechanisms by which inflammatory bowel diseases (IBD), metabolic syndrome and type 2 diabetes develop and persist [3], [29]–[31]. Furthermore, studies of IBD patient microbiota, reveal loss of potentially beneficial commensal microorganisms compared to control subjects [31]–[33]. At the same time, the role of intestinal bacteria in other inflammatory diseases such as type 1 diabetes is just beginning to be explored [34]. Motivated by the need to extend metagenomic analyses beyond the simple interrogation of gene catalogues, this study seeks to establish the potential of metatranscriptomics as a platform for investigating the mechanistic contributions of the gut microbiome on health and disease. Here we applied RNA-Seq on RNA preparations obtained from the intestinal contents of aged-matched non-obese diabetic (NOD) mice colonized with eight commensal bacteria. By focusing on a relatively simple community, our investigation centers on developing and optimizing experimental and analytical pipelines targeted specifically for metatranscriptomics.

## Results and Discussion

### Metatranscriptomic data from the intestines of non-obese diabetic (NOD) mice

To assess molecular protocols and computational analyses for Illumina-based metatranscriptomics, we sequenced RNA prepared from the flushed cecum and colon of age-matched NOD mice weeks prior to diabetes onset. The mice were the progeny of NOD animals that had been re-derived by embryo transfer into a completely germ-free environment and then colonized with an Altered Schaedler flora (ASF) containing eight known species [35], [36]: *Lactobacillus acidophilus*, *L. salivarius*, *Parabacteroides distasonis*, *Mucispirillum schaedleri*, three members of *Clostridium* cluster XIV and a relatively uncharacterized species of Firmicutes. All but *P. diastonis* (a member of the phylum Bacteroidetes) and *M. schaedleri* (a member of the phylum Deferribacteres) are members of the phylum Firmicutes. Although reference genome sequences of strains related to the two *Lactobacillus* spp. are available, we note that the two reference genomes for *L. acidophilus* (strains 30SC and NCFM) display considerable genomic variation, with 2037 and 1864 putative open reading frames (ORFs), respectively. Since the objective of many research groups is to apply metatranscriptomic methods to complex environmental and mammalian-host-associated bacterial communities that will exhibit diverse previously uncharacterized species, we selected the altered ASF colonized NOD intestinal samples, as a relatively simple pilot, to examine our ability to agnostically assign HTS reads to samples for which no reference genome is available.

We compared two RNA preparation methods: Qiagen RNAEasy (Qiagen, Valencia, CA), which selects for longer RNAs; and Invitrogen mirVANA (Invitrogen, Carlsbad, CA), which also purifies microRNAs. In addition, we examined the use of the Invitrogen RiboMinus Bacterial kit to selectively deplete rRNA, thereby enriching the high complexity mRNA. Twelve combinations of RNA preparation conditions were used and the samples were multiplexed and sequenced in a single lane of an Illumina Genome Analyzer IIx flow cell. Sequence reads have been made available for download from the National Center for Biotechnological Information (NCBI) Sequence Read Archive (SRA, http://www.ncbi.nlm.nih.gov/Traces/sra: Accession number: SRA051354). After filtering for HTS read quality, these samples produced 21.7 million single-end 76-nt reads (Table 1). rRNA sequences were identified through BLAST comparisons to an in-house database of rRNA sequences including sequences representative of all eight ASF species. Depending on sample, the use of the Qiagen RNAEasy kit alone resulted in 60–95% rRNA, while application of the Invitrogen RiboMinus kit to the same RNA samples reduced rRNA to 40–70% of total RNA. In contrast, the Invitrogen mirVANA protocol reduced rRNA to 30–40% of total RNA reads even in the absence of an rRNA depletion step.

**Table 1:** Sequence yields for 12 different sample preparations.

						Number of Reads Matching:					% of Non-Adaptor Reads Matching:		
Sample ID	Mouse	Anatomy	Method	Total Reads	Adaptor	rRNA	Mouse transcripts	Mouse genome	Bacterial transcripts (nt)	Bacterial transcripts (peptide)	Mouse transcripts/genome	rRNA	Bacterial transcripts
NOD501CecQN	501	Cecum	Qiagen	1,343,095	361,868	907,767	742	1,252	5,505	17,701	0.2	92.5	2.0
NOD501CecQY	501	Cecum	Qiagen*	2,071,165	893,317	458,168	14,562	22,322	31,722	305,991	3.1	38.9	28.2
NOD501ColQN	501	Colon	Qiagen	1,305,960	25,203	1,240,577	761	1,679	3,351	11,598	0.2	96.9	1.1
NOD502CecQN	502	Cecum	Qiagen	2,065,733	458,401	1,477,991	1,554	3,211	11,074	32,223	0.3	92.0	2.4
NOD502CecQY	502	Cecum	Qiagen*	1,409,494	688,903	514,693	5,156	5,860	11,649	61,766	1.5	71.4	9.7
NOD502ColQN	502	Colon	Qiagen	1,090,373	63,823	888,879	16,530	40,698	5,603	23,376	5.6	86.6	2.5
NOD503CecMN	503	Cecum	Invitrogen	2,073,136	360,711	726,392	70,744	191,760	36,855	288,111	15.3	42.4	18.1
NOD503CecQN	503	Cecum	Qiagen	1,566,243	244,192	1,227,864	890	2,449	8,065	27,483	0.3	92.9	2.4
NOD504CecMN	504	Cecum	Invitrogen	2,562,571	371,299	653,110	240,133	413,699	47,167	188,358	29.8	29.8	9.5
NOD504CecQN	504	Cecum	Qiagen	1,918,268	324,513	1,453,905	2,286	6,889	12,471	29,393	0.6	91.2	2.2
NOD504CecQY	504	Cecum	Qiagen*	2,626,735	1,012,199	1,152,379	16,372	14,597	27,756	130,581	1.9	71.4	9.3
NOD504ColQN	504	Colon	Qiagen	1,647,255	241,169	836,427	37,329	129,217	19,435	117,819	11.8	59.5	9.1

12 cecal and colon derived samples were prepared from four different NOD mice using a variety of RNA extraction protocols. Samples were multiplexed on a single Illumina sequencing run to generate 21.7 million sequences. Of these ∼1.5 million (∼10%) could be mapped to a known bacterial transcript either via BWA against bacterial genomes (nt) or via BLASTX against the protein non-redundant database (peptide). Use of the Invitrogen extraction kit resulted in the most consistent generation of a high proportion of bacterial transcripts.

‘*’ indicates the additional use of the RiboMinus kit.

### Phylogenetic analysis of metatranscriptomic rRNA sequence data

Because the mice used in this study were colonized with a defined set of bacteria, we could use the expected distribution of bacteria in the sample set to evaluate the utility of using relatively short rRNA reads to classify microorganisms. ASF comprises eight bacterial species representing three phyla (Firmicutes, Deferribacteres, and Bacteroidetes) and five families (Firmicutes: *Lachnospiraceae*, *Ruminococcaceae*, *and Lactobacillaceae*; Deferribacteres: *Deferribacteraceae*; and Bacteroidetes: *Porphyromonadaceae*). Although the three expected phyla were most often represented in the sequence dataset (>99% of reads), other phyla not representative of ASF species were detected, including Actinobacteria and Spirochaetes (Table 2). These aberrant classifications could have arisen from 1) small levels of other microorganisms in the germ-free colonies; 2) contamination of gut content samples following collection; or 3) misclassification. In both the cecum and colon, members of the phylum Bacteroidetes were the most abundant microorganisms, accounting for >85% of the SSU and LSU rRNA reads. However, at the family-level the expected and observed results differed drastically. Although all five ASF families were observed (Table 2), other related families were abundant in the dataset. For example, 40.2% of sequences were classified as *Bacteroidaceae*, rather than *Porphyromonadaceae* (19.6%). Similarly, 16.8% of sequences were classified as *Clostridiaceae*, rather than the related families *Lachnospiraceae* (17.5%) or *Ruminococcaceae* (0.12%). We interpret these results as evidence that short reads (50–76 nt) obtained by shotgun sequencing are inherently noisy markers of phylogeny; since shotgun reads may be generated from any sub-sequence of an rRNA transcript, reads originating from moderately to highly conserved gene segments may not contain sufficient numbers of unique characters to distinguish among low-level phylogenetic groups.

**Table 2:** Family-level distribution of small-subunit RNA sequences^1^.

		Animal:	501			502			503		504			
		Anatomy^2^:	Cec	Cec	Col	Cec	Cec	Col	Cec	Cec	Cec	Cec	Cec	Col
		RNA Prep^3^:	Qia	Qia	Qia	Qia	Qia	Qia	Inv	Qia	Inv	Qia	Qia	Qia
		RiboMinus^4^:	N	Y	N	N	Y	N	N	N	N	N	Y	N
Taxon^5^	Total Reads	ASF Species^6^												
Actinobacteria														
*Microbacteriaceae*	5620		0.4	0.1	0.2	0.3	0.2	0.2	0.4	0.2	0.3	0.2	0.1	0.1
Bacteroidetes														
*Bacteroidaceae*	994924		35.8	37.1	44.2	34.0	45.6	45.5	29.8	41.7	31.3	38.0	48.8	58.0
*Porphyromonadaceae*	485948	1	24.3	25.6	22.7	22.9	26.0	21.6	12.4	17.1	10.6	19.4	23.5	13.4
*Flavobacteriaceae*	16570		0.7	0.8	0.9	0.7	0.8	0.9	0.6	0.6	0.6	0.5	0.6	0.5
Deferribacteres														
*Deferribacteraceae*	1454	1	0.1	0.1	0.0	0.0	0.2	0.0	0.2	0.0	0.2	0.0	0.1	0.1
Firmicutes														
*Clostridiaceae*	415630		21.1	20.6	8.7	19.2	14.1	9.1	23.3	14.3	31.8	19.6	12.5	10.3
*Lachnospiraceae*	434676	3	13.2	11.0	17.9	17.6	9.8	17.2	27.5	21.4	19.4	18.1	11.1	13.8
*Ruminococcaceae*	3055	1	0.1	0.1	0.1	0.2	0.0	0.1	0.2	0.1	0.1	0.1	0.1	0.1
*Veillonellaceae*	43627		2.6	2.0	0.8	2.7	1.3	0.9	2.1	1.5	2.3	1.8	1.4	0.9
*Lactobacillaceae*	37903	2	0.7	0.9	2.9	1.1	0.7	3.0	1.2	1.8	1.2	1.2	1.0	1.8
Spirochaetes														
*Spirochaetaceae*	2764		0.1	0.1	0.0	0.1	0.2	0.0	0.5	0.1	0.4	0.0	0.1	0.1
Other reads:	31312		2544	753	4173	4881	632	2510	2793	4158	2242	3660	795	2171
Total reads:	2473483		251317	45213	245426	413910	50317	171243	153079	341282	129166	352836	105089	214605

1 Values are percents of total reads for sample.

2 Anatomic location of sample. Cec = Cecum contents. Col = Colon contents.

3 RNA extraction kit. Qia = Qiagen RNEasy. Inv = Invitrogen.

4 RiboMinus removal of bacterial rRNA from sample.

5 Phylum and family classifications of rRNA reads.

6 Number of Altered Schaedler Flora species belonging to bacterial family.

The RiboMinus protocol uses biotinylated antisense oligonucleotides with broad-specificities for bacterial small- and large-subunit rRNAs to selectively remove bacterial rRNA from samples. Because sequence heterogeneity and the kinetics of oligonucleotide hybridization with highly structured rRNA molecules potentially could result in biased rRNA depletion, we compared the phylogenetic distributions of mRNA and rRNA transcripts in depleted and non-depleted samples. Although ostensibly universal oligonucleotides were used to hybrid-capture and remove rRNA molecules, the frequencies with which bacteria were identified differed greatly following rRNA-depletion. For example, the phyla Bacteroidetes and Firmicutes were over- and under-represented following rRNA depletion of samples from animals 502 and 504 (Table 2). Thus, bacterial community composition within a sample cannot be reliably ascertained if prior removal of rRNA transcripts is performed.

### Mapping metatranscriptomic reads to transcripts requires a peptide-centric approach

Next we attempted to map the 5 million putative mRNA reads to known sequences. First we applied the sequence mapping tool, BWA [37], to filter and assign mouse-derived sequences (transcripts and genome). In total, 1,240,692 (∼24%) of the putative mRNA reads mapped to a mouse sequence. For most samples, we identified approximately twice as many mouse sequences through comparisons to the mouse genome compared to mouse transcripts, suggesting the majority of mouse derived sequences represent unspliced introns, 3′ or 5′ UTR's or other non-coding RNAs. In the absence of reference genomes for any of the eight ASF species, we then applied BWA to map reads to 1078 microbial genomes. Only 92,594 of the 5 million putative bacterial mRNA reads could be mapped by this approach. The less stringent sequence similarity search tool, megaBLAST, mapped only an additional 128,059 non-mouse reads. Since these tools rely on the identification of nucleotide matches, the low frequency of mapped reads presumably reflects the high level of sequence diversity that can occur even between different strains of the same species [38]–[40]. Such diversity may result from differences in codon usage that do not impact peptide sequences.

Consequently, we performed BLASTX comparisons (translated nucleotide v. protein) of putative mRNA reads against the protein non-redundant database (*protein-nr*). This strategy was more successful, with 1,234,400 of non-mouse-derived reads (32%) mapping to 237,570 unique bacterial transcripts. Most matches (>70–80% depending on sample) were high quality (≥85% sequence similarity over >65% of the read length - Figure S1). Notably, minimal overlap (<0.1%) occurred between reads matching bacterial transcripts through BLASTX comparisons and reads mapping to the mouse genome through BWA. Two samples (NOD501CecQN and NOD502CecQN) displayed considerably fewer high-quality mapped transcripts compared to the other 10 samples (48% and 62% matches >85% sequence similarity over >65% of sequence length respectively). These observations suggest that analysis of read matches may represent a useful quality control step. Of the other 10 samples, one prepared with the RiboMinus kit (NOD501CecQY) provided the highest proportion of mapped bacterial reads (28.2%), while the two Invitrogen mirVANA preparations also resulted in a relatively high proportion of reads mapped to a bacterial transcript (9.7 and 18.1% respectively - see Table 1).

**Figure 1:**
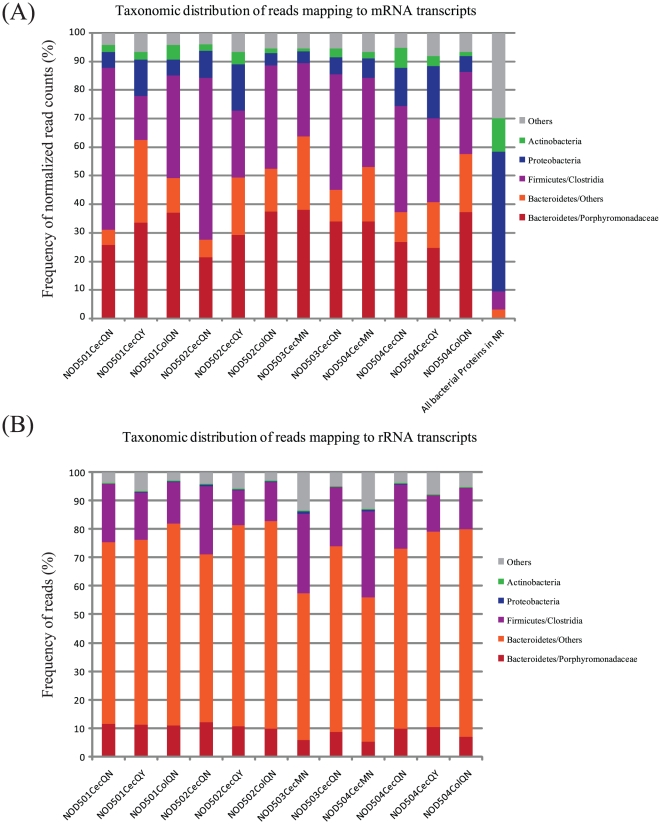
Phylogenetic distribution of transcripts mapped to known bacterial genes. (A) Distribution of mRNA transcripts. (B) Distribution of rRNA transcripts. Results are shown for the 12 independent samples described in Table 1. Consistent with 16S rRNA studies, the vast majority of identified mRNA transcripts derive from Parabacteroides, Bacteroides or Clostridial species.

Due to relatively short read lengths, a concern in these analyses is that matches to known transcripts are not meaningful. For a 25-residue peptide, allowing for only a single mismatch results in a bit score <50, while matches even with 60% identity result in E-values in excess of 10. To ensure that the bacterial transcripts identified through the BLASTX comparisons were consistent with the types of transcripts expected from the ASF bacteria, we examined the phylogenetic distribution of the assignable transcripts (Figure 1). To control for differences in transcript length [41], we converted raw read abundance to Reads Per Kilobase of transcript per Million reads mapped (RPKM - See Methods).

Consistent with the known ASF bacterial species, the majority of mapped transcripts derived from *Bacteroidetes* or *Clostridia* (Figure 1). Interestingly, many of the *Bacteroidetes* transcripts derive from the *Porphyromonadaceae* which includes the ASF species *Parabacteroides distasonis*. Furthermore, the phylogenetic distribution of the reads differed significantly from that of the entire set of bacterial proteins in *protein-nr*, which is dominated by *Proteobacteria*. Comparisons to the taxonomic distribution of the samples using the previously filtered small- and large-subunit rRNA reads revealed that, while the same three dominant taxa were observed in ORF and rRNA datasets, their relative representation in the datasets differed. For example, the representation of ‘Bacteroidetes/Others’ within the transcript dataset ranged from ∼5–28% in the mRNA dataset compared with ∼50–73% in the rRNA dataset. Although it is possible that this may reflect differences in relative activities between these taxa, these differences may also be a consequence of the relative paucity of mRNA sequence data that has previously been generated for this taxon. Alternatively as noted above, this discrepancy may also be driven at least in part by biases in the rRNA-depletion protocol. Finally, as noted above, inaccuracies in rRNA classifications may skew the comparisons of mRNA- and rRNA-based taxonomic distributions. For example, the mapped transcript dataset also contained sequences of actinobacterial or proteobacterial origin even though these were not represented within the ASF bacteria resident in the NOD mouse intestinal samples. We speculate that these transcripts may represent orthologs of *Bacteroidetes* or *Clostridium* genes for which no sequence representation exists in *protein-nr*. The dominance of expected taxa in the mapped transcript datasets suggests that they reflect the underlying distribution of mRNAs in the samples.

### Functional analysis of metatranscriptome datasets

RNA-Seq data derived from organisms with a reference genome can yield detailed gene-by-gene analysis of expression patterns. In contrast, for metatranscriptomic datasets, BLASTX-based mapping requires alternative methods that avoid the need to identify the precise source of a read. Instead, focus must shift to analyzing differences in expression levels of *functional classes* of genes [42]. Previous metatranscriptomics analyses have relied on broad functional categories such as those defined by the Clusters of Orthologous Groups (COG) of proteins database and the Gene Ontology (GO) resource [25], [43]. However, the generality of the functional vocabulary in these resources provide limited functional insights so that recent efforts have focused on specific functional classes such as gene families [26], [44]. Mapping *reads* to *transcripts* and, *transcripts* to *gene families*, reduces the need for gene-centric based approaches that require accurate transcript mappings to reference genomes. Reasoning that a gene family-based mapping approach may result in functional insights, we have analyzed the mouse metatranscriptome dataset in terms of gene families as well as three types of functional entities: COG functional categories; metabolic networks; and a protein-protein interaction network derived for *E. coli*.

#### Gene families

We applied the Markov clustering algorithm (MCL) to the 237,570 unique transcripts identified from our 12 samples to define 12,784 gene families on the basis of sequence similarity scores (see Methods). Summing the RPKM values for each transcript in a class was used to derive the relative abundance of each gene family. The size of the 500 gene families with the highest number of transcripts assigned did not correlate well with their relative expression (Figure S2). This suggests that gene family expression patterns did not merely reflect the abundance of transcripts identified in the initial mapping effort. Note here we use the term ‘expression’ to represent transcript abundance; as such it is important to consider that in addition to rates of transcription, ‘expression’ may also be driven through overall abundance of specific species. Table 3 shows the top 20 most represented gene families for the NOD503CecMN sample. The greatest correspondence in rankings of relative expression between samples occurred between samples that produced the largest number of reads that were reliably mapped to a known bacterial transcript (NOD504CecMN; NOD504ColQN, NOD501CecQY and NOD504CecQY). Eight of the top 20 most abundant families are annotated as hypothetical, of which three (GF8057, GF5525 and GF3794) are ranked in the top three across the majority of samples. GF8057, consisted of two members, both from *Parabacteroides* spp. (gi|154490247 and gi|218259679) while GF5525 contained five members, all from the order Clostridiales (gi|153812042, gi|154482409, gi|154482396, gi|154482831 and gi|169343363). Given their predicted relative expression, these hypothetical proteins merit further functional investigation. Among gene families that could be ascribed a putative function, eight were implicated in regulatory roles (e.g. GF1 - TonB-dependent receptor; GF3 - RagB/SusD domain protein; GF2 and GF9 - two component response regulators). The ability to map RNA-Seq data onto a defined set of gene families thus provides a surrogate for elucidating functional components from metatranscriptome data.

**Table 3:** Relative abundance of gene families.

Gene Family ID	# of Members	Description	Predominant Taxon	NOD501CecQN	NOD501CecQY	NOD501ColQN	NOD502CecQN	NOD502CecQY	NOD502ColQN	NOD503CecMN	NOD503CecQN	NOD504CecMN	NOD504CecQN	NOD504CecQY	NOD504ColQN
GF8057	2	Hypothetical	Parabacteroides	2	3735	2	2	583	2	1	2	1	3	44	1
GF5525	5	Hypothetical	Clostridiales	1	1	1	1	1	1	2	1	2	1	1	2
GF1	2934	TonB -dependent receptor	Bacteroides	8	2	14	10	3	6	3	6	4	13	5	3
GF4880	6	Hypothetical	Bacteroides	12	316	7	11	13	5	4	19	3	18	142	4
GF3	942	RagB/SusD domain protein	Bacteroides	69	4	24	20	6	20	5	16	8	29	10	6
GF782	66	Transposase	Various	394	5	38	868	15	135	6	179	14	289	138	12
GF12373	1	Hypothetical	Clostridiales	7	1303	22	12	17	22	7	5	61	9	33	8
GF843	61	RNA binding protein	Bacteroides	22	155	31	36	266	8	8	10	21	10	593	9
GF9210	1	Hypothetical	Neisseria	208	1783	1848	2395	4089	902	9	905	5	2684	316	10
GF2	1023	Two component response regulator	Various	165	13	59	9	18	97	10	123	12	63	168	13
GF3794	10	Hypothetical	Clostridiales	3	3	3	3	2	3	11	3	6	2	2	5
GF6	903	ABC transporter, ATP binding protein	Clostridiales	466	29	65	100	28	182	12	83	11	14	299	18
GF30	558	Anti-sigma factor	Bacteroides	275	8	91	157	21	173	13	198	18	76	18	20
GF8891	1	Hypothetical	Parabacteroides	18	255	5	14	952	4	14	27	16	23	567	17
GF129	265	ABC transporter, permease	Various	1166	15	1328	98	44	505	15	608	27	1214	23	53
GF9	818	Two component response regulator	Various	282	23	293	62	30	393	16	504	23	140	116	39
GF18	668	Beta galactosidase small chain	Various	124	24	98	50	29	91	17	58	17	47	79	30
GF82	350	RNA polymerase ECF-type sigma factor	Bacteroides	593	7	197	47	9	122	18	57	22	74	8	16
GF358	131	DNA-binding protein HU	Various	75	21	19	28	27	24	19	13	30	58	21	11
GF5	923	Short-chain dehydrogenase/reductase	Various	553	14	144	18	11	139	20	71	43	85	38	26

The 236,769 bacterial transcripts found to match reads were clustered into 13,278 gene families. The table indicates the relative abundance of each gene family as measured by summing RPKM values for each transcript in that family. Shown are the top 20 most abundant families in the NOD503CecMN sample together with the rank of abundance across all 12 samples. ‘Descriptions’ represent annotations associated with the transcripts according to definitions provided by Genbank. ‘Predominant taxon’ indicate the most frequent taxonomic group associated with the transcripts in the gene family.

#### COG functional categories

**Figure 2:**
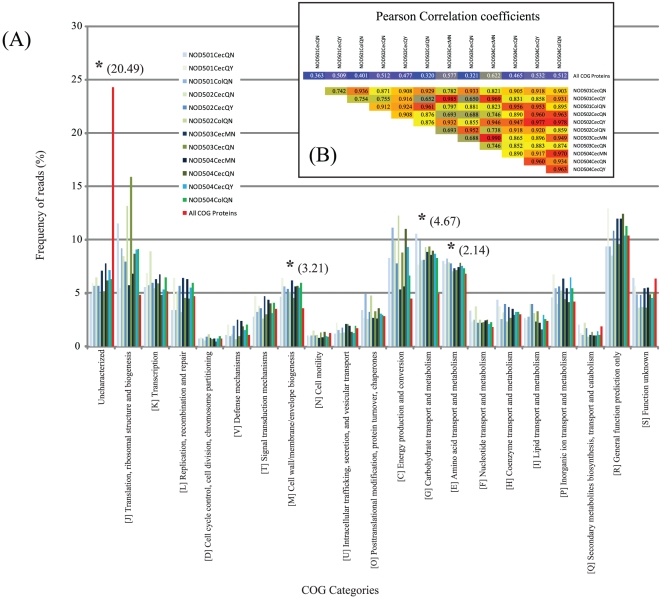
Distribution of COG functional annotations. (A) Distribution of COG functional annotations of reads mapping to known bacterial transcripts for the 12 samples analysed in this study. Also shown is the distribution of COG assignments for all proteins in the COG database (background). Asterisk's indicate COG categories with significant (Z-score>2) differences in relative frequency between samples and the background (Z-score is indicated). (B) Pearson correlation coefficients of frequency of reads assigned to each COG category across the 12 samples.

To examine the distribution of reads assigned to general functional categories, we assigned transcripts to COG categories on the basis of best BLAST matches to the COG database. RPKM values were then used to calculate the frequency of representation for each COG category. It should be noted in these analyses that as for the phylogenetic analyses, we observed consistency across samples (Figure 2). For many categories, the distribution for each sample reflected the underlying distribution of COG category assignment for all proteins in the COG database highlighting the limitations of using such a broad-category approach. Nonetheless, in addition to the ‘Uncharacterized’ category, three categories show significant (Z-score>2) differences between the samples and the COG library: [M] - Cell wall/membrane/envelope biogenesis; [C] Energy production and conversion; and [G] Carbohydrate transport and metabolism. The intestinal microbial transcriptome enrichment of these categories may therefore reflect exploitation of carbohydrates for the production of biomass. On the other hand, in these analyses it is important to note that observed differences represent relative (as opposed to absolute) enrichments that could simply reflect decreases in other categories (e.g. ‘Uncharacterized’).

#### Metabolic networks

**Figure 3:**
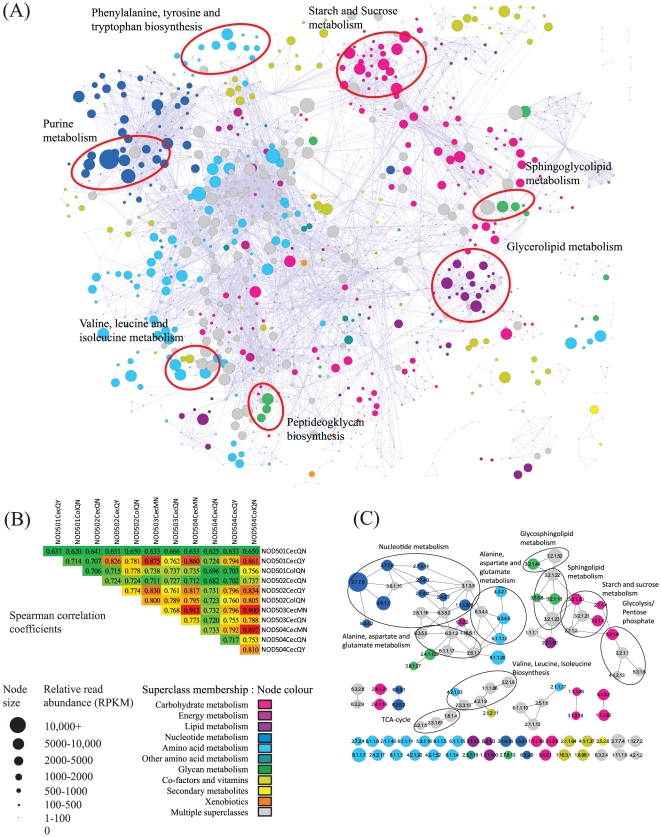
Metatranscriptome data mapped in the context of a global metabolic network. (A) Network map highlighting metabolic enzyme expression for reads obtained from the NOD503CecMN sample. Size of node indicates relative expression. Colour of node indicates functional category of enzyme as defined by KEGG superclasses (see key for details). Several example pathways are indicated. (B) Spearman rank correlation coefficients of relative enzyme expression across the 12 samples. In general there is a high degree of consistency in enzyme expression across samples. (C) Top 100 expressed enzymes in the NOD503CecMN sample. For three subnetworks, links are apparent that extend beyond the boundaries of KEGG defined pathways.

We next examined functional representation within our datasets by representation of metabolic pathways. Previous analyses have focused on KEGG representations of metabolic pathways [3], [7] which may not capture alternative pathways that utilize different suites of enzymes to achieve similar functions [45]. Here we adopted a network-based approach to identify neighbourhoods of functionally related genes (Figure 3A). From these analyses we identified several pathways that were well represented across datasets. These pathways included starch and sucrose metabolism, amino acid biosynthesis, glycerolipid metabolism, peptidoglycan biosynthesis as well as components of purine metabolism. These latter components are largely associated with the production of RNA and DNA from purine precursors (data not shown). Reassuringly, pathways specific to eukaryotes such as N-glycan biosynthetic pathways [46], were poorly represented across our datasets. As for the COG analyses, we found a high degree of correlation in the expression of enzymes that were inferred across samples. Perhaps surprisingly, high correlations were observed between cecum and colon-derived samples despite the presumably different environment to which the microbes in these sites were exposed (Figure 3B).

To demonstrate how a network-based approach may transcend traditional KEGG defined pathways, we constructed a subnetwork of enzymes for the top 100 expressed enzymes (in terms of RPKM) represented in the NOD503CecMN dataset (Figure 3C). Within this network we identified a link between enzymes in the valine, leucine and isoleucine biosynthetic pathway, which appear to feed acetyl-CoA, produced from the synthesis of alpha-isopropylmalate by isopropylmalate synthase (EC:2.3.3.13), into part of the TCA cycle. In a second example, components of glycosphingolipid, sphingolipid, starch and sucrose metabolism are linked to glycolysis and the pentose phosphate pathway, suggestive of the breakdown of compounds associated with the former to feed glycolysis and the production of ribose. In a final example, components of nucleotide metabolism are linked to alanine, aspartate and glutamate metabolism through the production of adenylosuccinate by adenylosuccinate synthase (EC:6.3.4.4). Together these pathways are indicative of the biochemical routes adopted by the microbiome driving the production of biomass.

**Figure 4:**
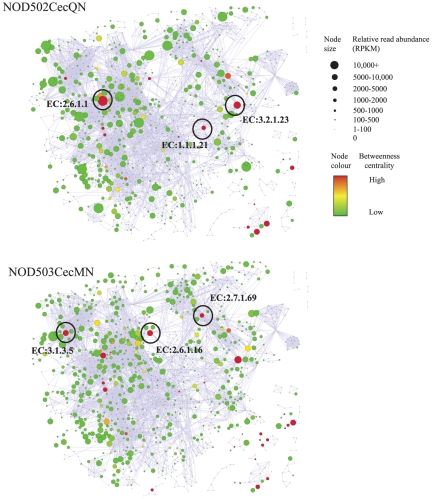
Samples display subtle differences in expression of enzymes of high betweenness centrality. For each enzyme in the network, betweenness centrality was calculated and mapped to node colour. Between the two samples we identify differences in the expression of nodes of high betweenness, suggesting an altered reliance on pathway flux within the network.

Adopting a network approach facilitates the identification of nodes that mediate important roles within the network. Betweenness centrality is a metric that assesses the importance of the node to the network through determining the proportion of shortest path lengths that pass through that node [47]. Focusing on nodes of high betweenness centrality, we observe differences between samples (Figure 4). For example, although both the NOD502CecQN and NOD503CecMN samples show similar levels of expression of beta-galactosidase (EC:3.2.1.23), the former has higher levels of aldehyde reductase (EC:1.1.1.21) and aspartate transaminase (EC:2.6.1.1). On the other hand, the NOD503CecMN sample has higher levels of 5′-nucleotidase (EC:3.1.3.5); glucosamine–fructose-6-phosphate aminotransferase (EC:2.6.1.16) and protein-*N* ^π^-phosphohistidine—sugar phosphotransferase (EC:2.7.1.69). Such differences may indicate subtle changes in the reliance of these key enzymes for directing flux within the network.

Consistent with previous metagenomic analyses, these studies have highlighted the importance of metabolic activities involved in the production of biomass. More importantly, the use of the network for mapping metatranscriptomic datasets shows how groups of functionally related enzymes, differentially expressed across samples, can be readily identified.

#### E. coli protein-protein interaction networks

**Figure 5:**
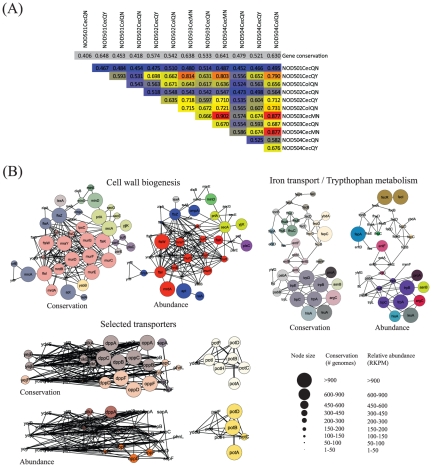
Metatranscriptome data mapped in the context of a global *E. coli* protein interaction network. (A) Spearman correlation coefficients of abundance of reads mapping to *E. coli* homologs across samples. (B)–(D) Comparison between conservation, as defined by number of bacterial genomes in which a putative ortholog has been identified and relative expression of *E. coli* homologs for three selected subsystems derived from the transcriptome data for a single sample (NOD503CecMN). Subsystems are defined from a previously generated high quality protein-protein interaction network [53]. Colours of nodes indicate genes involved in common functional modules. Size of nodes indicate either number of genomes or relative expression in terms of RPKM (largest = >900 genomes/transcripts). (B) Proteins involved in cell division and cell wall biogenesis. (C) Proteins involved in iron transport and tryptophan metabolism. (D) Proteins involved in select transport pathways - note of the ones shown, despite many being highly conserved, only components involved in spermidine transport (PotA-D) are abundant within our sample.

Compendia of physical and functional interactions are now available for bacteria that can be exploited as scaffolds onto which RNA-Seq data may be mapped [48]–[53]. Although the focus of these datasets on *E. coli* will undoubtedly preclude the identification of systems specific to particular taxa (e.g. Bacteroides and/or Clostridiales), many processes such as cell wall biogenesis, transcription and translation are well conserved throughout bacteria. We may therefore expect these analyses to yield insights into the activity of basic core processes. First, we identified *E. coli* homologs of each transcript on the basis of InParanoid-derived relationships [54]. The relative abundance of each *E. coli* gene was then generated from the sum of RPKM values of the transcripts that map to each gene. Correlation across samples varied (Spearman correlation coefficients 0.45–0.90) but was greatest between samples with the most mapped reads (Figure 5A). Within a single sample, *E. coli* gene abundance ranged from 1 to 12,936 reads, a dynamic range of four orders of magnitude with the most highly expressed for eight samples being *FepA* (an outer membrane receptor associated with the ferric enterobactin transport system). As noted above, the focus on *E. coli* suggests that the relative expression of each gene may correlate with its relative level of conservation. This might imply that our mapping simply reflects underlying conservation biases and indeed we note some correlation between expression and conservation (Figure 5A). However, mapping these data onto a high quality protein-protein interaction template readily identifies components of several diverse bacterial processes with expression profiles that do not correlate with conservation (Figure 5B–D). For example, we identify both components that are poorly expressed but well conserved (e.g. murC, murF and murG involved in cell wall biogenesis) as well as components that are well expressed but poorly conserved (e.g. fepA, fecI and fecR involved in iron transport). Furthermore, for a selected group of transporters, although many are well conserved, we note that only those components associated with spermidine transport (potA, potB, potC and potD) are also well expressed, suggesting a biologically meaningful role for this transport function within our samples. Finally, it is important to highlight a recent metatranscriptomic study that showed expressed genes to be significantly more evolutionarily conserved than non-expressed genes [55], demonstrating the biological relevance of the relationship between conservation and expression.

### Summary and Future Perspectives

Here we have shown that in the absence of reference genomes, RNA-Seq technology may be applied to environmental samples. Despite the low statistical significance associated with sequence matches, the phylogenetic assignments of the mapped transcripts were skewed towards those expected of ASF-colonized mice, suggesting that this approach is biologically meaningful. By placing transcripts into *functional classes*, functional insight can be gained either on the basis of associated annotations of e.g. representative gene families, or through more sophisticated systems-based approaches that exploit network relationships. In this study, we note several areas that would benefit from recent technological developments as well as the development of new algorithms. First, we were able to map only a limited number of reads to known bacterial mRNA transcripts. The development of paired-end reads allied to increases in read length associated with the Illumina HTS platform should enhance homolog detection. In addition, given sufficient depth of sequencing coverage, it may be possible to combine reads into larger ‘contigs’ that would increase the information content of the reads. However, currently available short-read assembler algorithms [56], [57] have not been optimised for assembling metatranscriptomic data and our initial attempts applying the Velvet short read assembler [57] resulted in few contigs longer than individual reads (data not shown). A significant challenge in the field is the expected shallow coverage of transcripts found in samples, such as mammalian commensal communities, containing hundreds or thousands of bacterial species, and the need to avoid the generation of chimeric contigs. An interesting question thus arises as to what is the depth of sequencing required to inform on a specific community. Factors to consider include diversity of species associated with the community as well as the size of their genomes. On the other hand, while it is clear as sequencing costs continue to fall, that high sequencing depths will become readily attainable and further development of analytical pipelines such as those presented here will be critical to avoid major bottlenecks in evaluation of these datasets.

At present, there is no combination of metagenomic analyses that can accurately and comprehensively define the composition and function of a complex bacterial community. This limitation reflects a nascent field characterised by a paucity of annotated bacterial genomes, the inability to culture most environmental species, error from PCR primer bias and high throughput sequencing and reliance on models for mapping sequence reads. For example, surveys of 16S rRNA gene sequences between samples may be functionally informative if the abundances of bacterial groups differ significantly. However, closely related bacterial strains that differ in their gene expression between sets of environments or disease states would not be detected by this approach. While current computational tools for metatranscriptomics analysis cannot yet assign genes to unique species, we show that this approach has the potential to reveal the functional architecture of all genes expressed by a defined community. Moving to the future, we envisage that a complete characterization of the organization and interrelationships of microbial communities will require the integration of several complementary datasets including: metagenomics, metatranscriptomics, 16S rRNA gene surveys as well as proteomics and metabolomics. On the other hand, these studies will require considerable investment in resources; consequently pilot studies such as the one presented here are essential if we are to address issues of feasibility before committing to large scale investments.

## Materials and Methods

### Housing and handling of mice

The mice used in the RNA sequencing study were born from NOD dams that were the progeny of mating pairs born in a completely germ-free environment following axenic (sterile) embryo transfer into germ-free pseudo-pregnant females. The germ-free status of mice in the facility is maintained by housing in flexible-film isolators and monitored by qPCR analysis of fecal DNA preparations using pan-specific bacterial 16S primers, and culture of cecal contents under anaerobic (blood agar) and aerobic (Luria broth) culture conditions. In addition DNA staining of cecal contents with Sytox green is used to confirm the bacterial absence. The absence of parasites, bacteria and virus contamination was independently confirmed quarterly by shipment of sentinel mice for analysis at a commercial facility.

Once GF status was confirmed as described, GF NOD mice were colonized by co-housing with gnotobiotic mice colonized with defined cultured bacterial species (Altered Schaedler's flora; ASF - [35]) which were prepared in the laboratory from cloned bacteria using sterile technique. The ASF consists of *Lactobacillus acidophilus* (ASF 360), *Lactobacillus murinus* (ASF 361), *Bacteroides distasonis*, (ASF 519), *Mucispirillum schaedleri* (ASF 457), *Eubacterium plexicaudatum* (ASF 492), a Fusiform-shaped bacterium (ASF 356) and two Clostridium species (ASF 500, ASF 502). These ASF-colonized gnotobiotic mice were then bred in isolators to ensure no additional species were introduced. The presence of the ASF species was confirmed by species-specific bacterial qPCR [58].

### Preparation of samples and sequencing

Four gnotobiotic NOD mice colonized with ASF were sacrificed at 12 weeks of age. The intestinal tract was immediately removed and splayed open through a longitudinal incision. Luminal contents from the small intestine (SI), cecum, and colon were collected individually, by scraping biomass from intestinal wall with a sterile scalpel. Specimens were placed in separate microcentrifuge tubes, frozen at −80C, and shipped to the laboratory of Dr. Frank on dry ice. Upon thawing, approximately 30 mg luminal biomass was suspended in two volumes (mass/vol) of phosphate buffered saline (pH7.4), producing a 100 µl slurry of biomass. Two RNA extraction protocols were compared: 1) Cell lysis in RNAzol B Reagent (IsoTex Diagnostics, Inc., Friendswood, TX) followed by RNA purification using the RNeasy kit (Qiagen Inc., Valencia, CA [59]); and 2) Cell lysis in mirVana™ lysis/binding buffer (Invitrogen, Inc. Carlsbad, CA) followed by RNA purification using the mirVana™ kit (using the total RNA procedure). In both procedures, 100 µl of sample slurry was added to 1 ml of lysis buffer and ca. 250 mg of 0.1 mm zirconium beads (Biospec Inc. Bartlesville, OK). Specimens were disrupted by bead-beating for 2 min in a Mini-Beadbeater-8 (Biospec Products Inc, Bartlesville, OK) followed by purification following the manufacturers' protocols. Furthermore, 3 µg aliquots of three total RNA samples (from the Qiagen-based protocol) were depleted of bacterial rRNA molecules by application of the RiboMinus™ Transcriptome Isolation Kit and RiboMinus™ Concentration Module (Invitrogen, Inc. Carlsbad, CA). Aliquots of total RNA were separated by 1% agarose/TBE gel electrophoresis and visualized by ethidium bromide staining. RNA prepared from cecum and colon was dominated by distinct rRNA bands, whereas samples prepared from SI appeared degraded. Consequently, SI samples were not subjected to sequencing. A total of 12 RNA samples were submitted for next-generation sequencing (Table 1).

Sequencing was performed with the Illumina Genome Analyzer IIx (GaIIx) platform at the Center for Advanced Genomics (TCAG - Hospital for Sick Children). After deconvolution of the barcodes used for multiplexing, 21,680,028 76 bp reads were generated on a single flow cell. Reads are available for download from the National Center for Biotechnology Information (NCBI) Sequence Read Archive (SRA, http://www.ncbi.nlm.nih.gov/Traces/sra: Accession number: SRA051354). Poor quality bases were removed by iterating a 5 nt window across the 5′ and 3′ ends of each sequence and removing nucleotides in windows with a mean quality score <20; iteration was stopped when the mean quality score was >20. Adaptor sequences were removed using Cross_match (http://www.phrap.org) to search a database of Illumina adaptor sequences. Following trimming and adaptor removal, reads with lengths less than 50 nt were discarded. Due to the poor performance obtained from applying the Ribosomal Database Project (RDP) classifier [60] to relatively short reads, putative rRNA transcripts were identified by BLAST sequence similarity searches (bit score >50) against an in-house database of rRNA sequences constructed from the All-species Living Tree Project SSU database [61], supplemented with ASF SSU sequences [35] and 5S and LSU sequences [62] representative of intestinal microbes. Blast hits with bit scores >50 were removed from mRNA datasets. In all, 5,096,278 reads of putative mRNA transcript origin were identified and subjected to further analyses.

### Sequence processing

To identify potential host contaminants, putative mRNA transcripts were mapped to: 1) a database of mouse derived transcripts (Ensemble release V.59 – http://www.ensembl.org); 2) the mouse genome; and 3) a database of 1078 bacterial genomes downloaded from the NCBI (June, 2010), using the software tool BWA [37]. Subsequent searches were performed using BLASTX [63] against the set of bacterial proteins extracted from the protein non-redundant database. To account for expression bias due to transcript length, each sample transcript expression was normalized to provide values of Reads Per Kilobase of transcript per Million reads mapped (RPKM - [41]) using the formula:
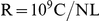
where C = number of reads that could be mapped in that sample to the specific bacterial transcript, L = the length of the transcript and N = total number of reads that could be mapped to bacterial transcripts in that sample.

### Assigning functional classes

To generate gene family assignments for each transcript, we performed an all-vs-all BLAST search of the 236,769 unique transcripts identified from our 12 samples (E-value<10^−5^). The Markov clustering algorithm (MCL - [64]) was then applied using an inflation parameter of 2.6 to place each transcript into one of 13,278 gene families. For each sample, the relative expression of each gene family was derived from the sum of RPKM values for each transcript associated with that gene family. COG category [65] assignments were performed through BLAST-based similarity searches to identify the closest matching sequence in the COG database (E-value<10^−3^). Enzyme classification (EC) assignments were assigned by performing a BLASTP (E-value<e^−10^) search of the 246,538 transcripts against a database of 127,478 enzyme proteins annotated by SwissProt Version 57.0 [66]. A slightly more stringent E-value is used here to reduce the number of false positives that arise when sequence similarity is used for enzyme classification purposes [67]. Metabolic networks were constructed as previously described [68]: enzymes (EC numbers) are represented as nodes and substrates connecting two enzymes are represented as edges in the network. Enzyme-substrate relationships were inferred from KEGG [28]. *E. coli* homolog mapping was performed through BLAST-based similarity searches to identify the closest matching sequence in the set of *E. coli* K12 transcripts (E-value<10^−3^). The relative abundance of each COG category, EC number and *E. coli* homolog was derived from the sum of RPKM values for each transcript that maps to the specific category, number or homolog in question. The relative conservation of *E. coli* genes was generated through the systematic identification of putative orthologs of each *E. coli* gene in the set of 1078 bacterial genomes using the tool InParanoid [69]. Network metrics were computed using the BGL library in MatLab (http://www.mathworks.com/matlab).

## Supporting Information

Figure S1 **Distribution of sequence matches from BLASTX searches against a set of bacterial transcripts.** For each sample, we show the frequency of reads that have a match to a known bacterial transcript at a specific threshold of % sequence identity and % of read length.
(EPS)

Figure S2 **RPKM values of the top 500 largest gene families.** The graphs indicate the RPKM values associated with each gene family as a function of the number of members associated with that family. For each sample we observe only weak correlation between the size of the family and its relative expression within the datasets.
(EPS)
